# Integrating Multiple Analytical Datasets to Compare Metabolite Profiles of Mouse Colonic-Cecal Contents and Feces 

**DOI:** 10.3390/metabo5030489

**Published:** 2015-09-11

**Authors:** Huawei Zeng, Dmitry Grapov, Matthew I. Jackson, Johannes Fahrmann, Oliver Fiehn, Gerald F. Combs

**Affiliations:** 1 Grand Forks Human Nutrition Research Center, Agricultural Research Service, United States Department of Agriculture, Grand Forks, ND 58203, USA; E-Mails: matthew.jackson@ars.usda.gov (M.J.); gerald.combs@ars.usda.gov (G.C.); 2 West Coast Metabolomics Center, University of California, Davis, CA 95616, USA; E-Mails: dgrapov@ucdavis.edu (D.G.); jfahrmann@ucdavis.edu (J.F.); ofiehn@ucdavis.edu (O.F.)

**Keywords:** metabolite, mass spectrometry, colon, cecal contents, feces

## Abstract

The pattern of metabolites produced by the gut microbiome comprises a phenotype indicative of the means by which that microbiome affects the gut. We characterized that phenotype in mice by conducting metabolomic analyses of the colonic-cecal contents, comparing that to the metabolite patterns of feces in order to determine the suitability of fecal specimens as proxies for assessing the metabolic impact of the gut microbiome. We detected a total of 270 low molecular weight metabolites in colonic-cecal contents and feces by gas chromatograph, time-of-flight mass spectrometry (GC-TOF) and ultra-high performance liquid chromatography, quadrapole time-of-flight mass spectrometry (UPLC-Q-TOF). Of that number, 251 (93%) were present in both types of specimen, representing almost all known biochemical pathways related to the amino acid, carbohydrate, energy, lipid, membrane transport, nucleotide, genetic information processing, and cancer-related metabolism. A total of 115 metabolites differed significantly in relative abundance between both colonic-cecal contents and feces. These data comprise the first characterization of relationships among metabolites present in the colonic-cecal contents and feces in a healthy mouse model, and shows that feces can be a useful proxy for assessing the pattern of metabolites to which the colonic mucosum is exposed.

## 1. Introduction

The gastrointestinal tract, which is the site of the host’s first encounter with nutrients, microorganisms and other bioactive factors in foods, hosts a complex community of microorganisms. The role of that community in affecting health is becoming increasingly evident. Research has shown that differences in the nature of the gut microbiome are associated with risk to obesity [1], colon cancer [2], and type 2 diabetes mellitus [3]. Such effects would appear to involve the production by the gut microbiome of metabolites used as substrates and/or signaling molecules by mucosal epithelial cells.

Metabolites represent molecular read-outs of cells. The pattern of metabolites produced by the gut microbiome comprises a phenotype indicative of the means by which that microbiome affects the gut. Such metabolic phenotypes can be characterized using metabolomic approaches, which employ the capacities of tandem liquid/gas chromatography-mass spectrometry to profile metabolites.

Two major platforms are available for metabolomic analyses: gas chromatography, time-of-flight mass spectrometry (GC-TOF) and liquid chromatography quadrapole, time-of-flight mass spectrometry (LC-Q-TOF). The former is useful for the analysis of thermally stable volatile compounds, typically >600 daltons, and yields high separation efficiency and reproducible retention times and mass spectra. The latter does not require derivatization, and is amenable for the analysis of polar and high molecular weight metabolites [4,5]. Combining data produced by both platforms offers comprehensive insight into actual metabolomic phenotypes. 

Interrogating high-dimensional metabolomic data calls for several analytical tools: multiple hypothesis testing with false discovery rate (FDR) adjustment, hierarchical clustering, principal components analysis (PCA), partial least squares (PLS), biochemical pathway enrichment and network analysis [6,7]. With these tools, difficult and complex analyses of large amounts of data can be more easily accomplished and interpreted.

Little is presently known about the metabolic phenotypes of the gut microbiome nor whether those can be estimated by analyzing feces, a more readily accessible specimen, particularly in clinical contexts. Therefore, we compared the metabolic profiles of the colonic-cecal luminal contents and recently collected feces of C57BL/6 mice fed a standard chow-type diet. These baseline data can inform studies of the host-gut environment and the whether fecal analyses may be informative in such studies.

## 2. Results and Discussion

### 2.1. Metabolomic Comparisons of Sample Types

**Figure 1:**
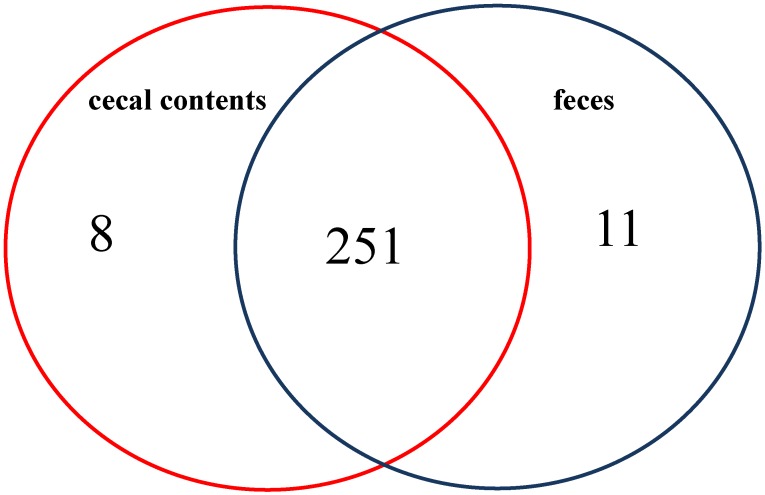
Overview of significant differences in metabolite profiles between colonic mucosal, colonic-cecal content and feces sample type comparisons.

A total of 270 metabolites were identified in the colonic-cecal contents and feces. Of these 251 (93%) metabolites were found in both types of specimen. Only 19 showed metabolites unique patterns (eight found only in colonic-cecal contents; 11 found only in feces) (Figure 1); however, 115 metabolites showed significantly different relative abundances in colonic-cecal contents *vs*. feces (p_adj_ < 0.05) (Figure 1, Table S1). Metabolic pathway enrichment analysis revealed that these 115 metabolites fell into 21 biochemical pathways (as defined by Kyoto Encyclopedia of Genes and Genomes, KEGG [8]), which were mainly related to amino acid, lipid metabolism, rare amino acids, cofactors, vitamins, signaling molecules, nitrogen, energy, and human disease/cancer.

In this study, the chow diet contained 15.5% fiber (including crude and detergent fiber, e.g., cellulos, hemi-cellulose and lignin according to product data analysis (LabDiet cat#5015, St. Louis, MO). Certain dietary fibers are fermentable, and can alter gut microbiota composition. It is known that the gut microbiota is exposed to substrates that have escaped digestion in the upper gastrointestinal tract, or have been released into the lumen in the form of mucous and sloughed cells [9,10], and that their fermentation produced short chain fatty acids (SCFAs) [10]. Accordingly, the metabolite patterns we found demonstrate the presence of metabolic pathways involved the metabolism of lipids and amino acids.

### 2.2. Metabolites Unique To Colonic-Cecal Contents or Feces

Certain metabolites were found only in colonic-cecal contents or feces. Eight found only in colonic-cecal contents were related mostly to the metabolism of lipids, amino acids and nucleotides as well as to digestion and carcinogenesis (Table 1). Eleven metabolites found only in feces were related to the metabolism of lipids, amino acids and some vitamins, as well as glycan biosynthesis, digestion system and carcinogenesis (Table 1). The relative abundances of these unique metabolites were included in (Table S1).

**Table 1:** Unique metabolites and their respective biochemical pathways in colonic-cecal contents and feces.

Colon-cecal contents	Feces
Unique metabolites	
1-Hexadecanoyl-2-octadecadienoyl-sn-glycero-3-phosphocholine	1H-Indole-3-carboxylic acid
L-Alanyl-L-norleucine	1-Myristoyl-sn-glycero-3-phosphocholine
Leu-Val	1-Octadecanoyl-sn-glycero-3-phosphocholine
N-Palmitoylsphingosine	Betaine
Palmityl-L-carnitine	L-Carnitine
Pregnan-20-one, 17-(acetyloxy)-3-hydroxy-6-methyl-, (3b,5b,6a)-(A)	Nicotinamide adenine dinucleotide (NAD)
3a,12b-Dihydroxy-5b-cholanoic acid-(A)	Oxyquinoline
Deoxythymidine monophosphate (dTMP)	Pregnan-20-one, 17-(acetyloxy)-3-hydroxy-6-methyl-, (3b,5b,6a)-(B)
	13-hydroxy-9Z,11E-octadecadienoic acid
	3a,12b-Dihydroxy-5b-cholanoic acid-(B)
	UDP-N-acetyl-D-galactosamine
Respective biochemical pathways	
Ether lipid metabolism	Primary bile acid biosynthesis
Glycerophospholipid metabolism	Tryptophan metabolism
Metabolic pathways	Biosynthesis of secondary metabolites
Choline metabolism in cancer	Ether lipid metabolism
Valine, leucine and isoleucine degradation / biosynthesis	Glycerophospholipid metabolism
Sphingolipid metabolism	Metabolic pathways
Bile secretion	Choline metabolism in cancer
Fatty acid degradation	Glycine, serine and threonine metabolism
Steroid hormone biosynthesis	ABC transporters
Secondary bile acid biosynthesis	Bile secretion
Pyrimidine metabolism	Fatty acid degradation
	Folate biosynthesis
	Nicotinate and nicotinamide metabolism
	Quinolines
	Phenylalanine, tyrosine and tryptophan biosynthesis
	Steroid hormone biosynthesis
	Biosynthesis of unsaturated fatty acids
	Linoleic acid metabolism
	Secondary bile acid biosynthesis
	Mucin type O-Glycan biosynthesis
	Amino sugar and nucleotide sugar metabolism
	Mucin type O-Glycan biosynthesis

Their largely similar metabolite profiles indicate the presence of the same functional pathways in both colonic-cecal contents and feces. Only 7% of metabolites were unique to colonic-cecal contents or feces. These may have utility as signature molecules for these specimens. The presence only in colonic-cecal contents of peptides (Leu-Val, L-Alanyl-L-norleucine) and metabolites of bile acids, steroids, lipids and nucleotides (1-Hexadecanoyl-2-octadecadienoyl-sn-glycero-3-phosphocholine; Palmityl-L-carnitine; pregnan-20-one,17-(acetyloxy)-3-hydroxy-6-methyl-(3b,5b,6a)-(A); 3a, 12b-dihydroxy-5b-cholanoic acid-(A); N-palmitoylsphingosine, deoxythymidine monophosphate) is consistent with the fact that the complete peptide-breakdown only occurs in colonic segment [11,12,13,14], and cecum also plays a critical role in bile secretion and steroid and lipid metabolism [15]; whereas, the presence only in feces of metabolites of amino acids, bile acids and lipids (1H-Indole-3-carboxylic acid; betaine; oxyquinoline, 1-Myristoyl-sn-glycero-3-phosphocholine, 1-Octadecanoyl-sn-glycero-3-phosphocholine, L-Carnitine, 3a,12b-Dihydroxy-5b-cholanoic acid-(B)), some fats, glycans and heterocylic compounds (NAD; 13-hydroxy-9Z, 11E-octadecadienoic acid; UDP-N-acetyl-D-galactosamine; oxyquinoline) is consistent with bacterial fermentation in the distal colon [9,16]. However, we cannot be sure the extent to which the latter metabolites may have been produced under aerobic conditions after defecation.

### 2.3. Metabolite Patterns and Networks

**Figure 2:**
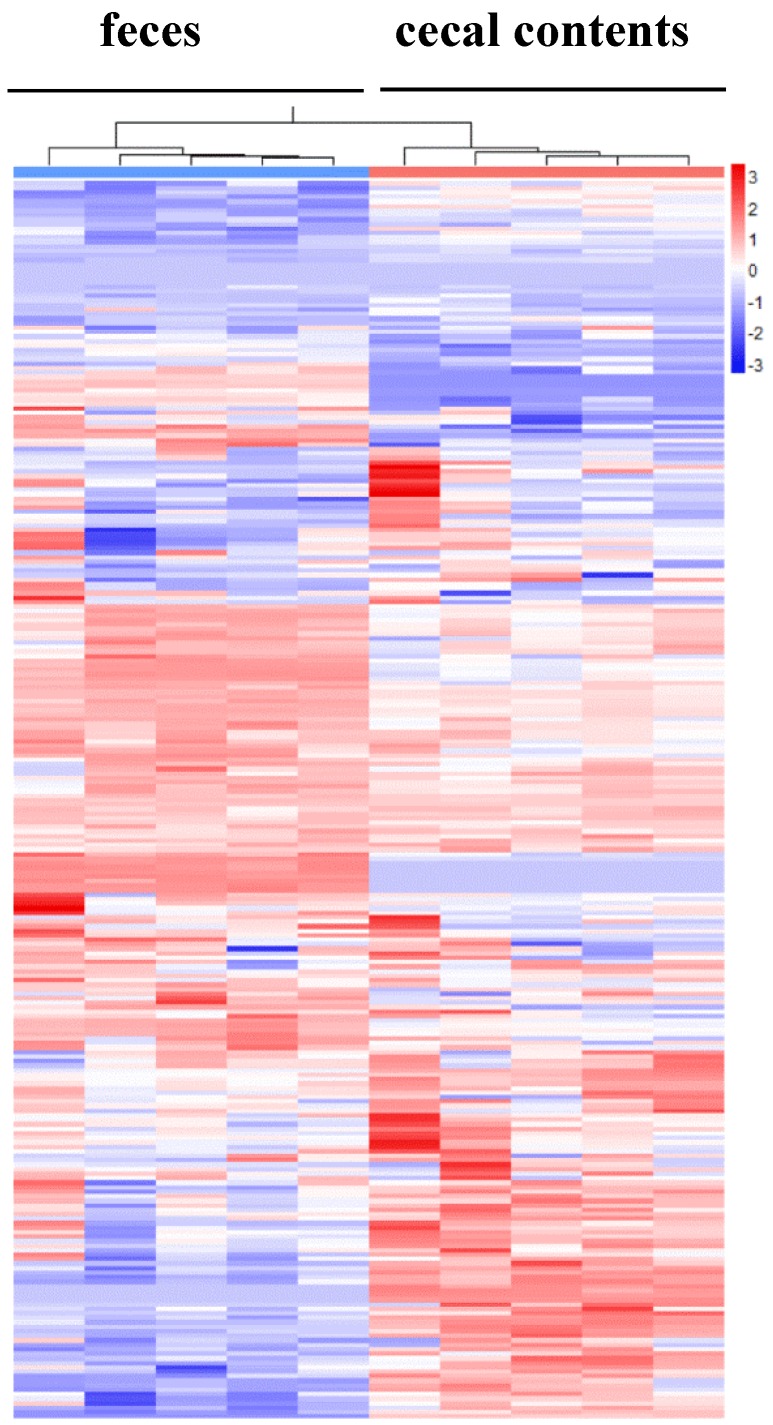
The heatmap displays relative increase/decrease of metabolite contents and their similarities between individual samples. In these visualization columns represent samples and rows variables, and hierarchical cluster analysis (HCA) was used to group samples and metabolites based on similarities in auto-scaled values and correlations, respectively. Cluster identities represent differing experimental biological or analytical variability; colors (red, relative increase; blue, relative decrease).

**Figure 3:**
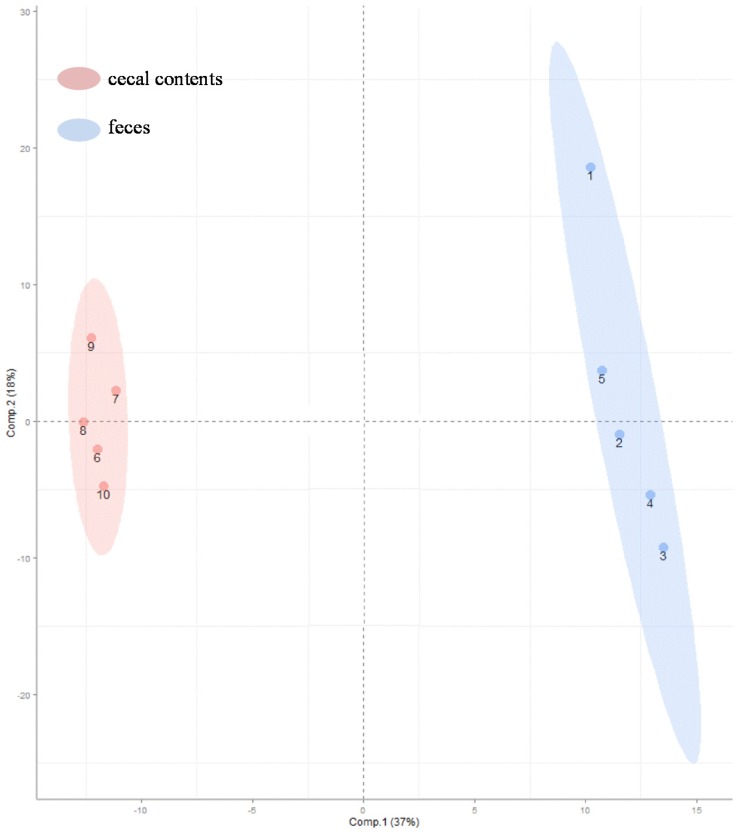
Orthogonal signal correction partial least squares discriminant analysis (O-PLS-DA) was used to generate a multivariate classification model showing the relationship of the variance (in metabolites) and sample types (colonic-cecal content and feces). X and Y axis are O-PLS-DA sample scores for the first two latent variables. Color codes: pink, colonic-cecal contents; blue, feces.

Comparisons of the relative abundances of the 270 metabolites found in these specimens are shown as a heat map [17] (Figure 2). As the smaller bracket (at the top of Figure 2) represents the higher similarity in abundance between individual samples, the data show that samples within a given sample type (e.g., cecal contents or feces) shared very similar patterns (Figure 2). The cecal contents and feces also shared an overall similar metabolite profiles with only a few distinct patterns because feces were downstream-products of colonic-cecal contents via colonic fermentation (Figure 2, Table S1). Orthogonal signal correction partial least squares discriminant analysis (O-PLS-DA) [18] was used to develop a multivariate classification model for these two sample types. The two latent variable (X and Y axis) for an O-PLS-DA model (Figure 3), can be used to evaluate within- and between-group similarities between samples, and the smaller values of latent the more similar [6,18]. As latent variables of metabolites in cecal contents were smaller than that of feces (Figure 3), it suggests that inter individual variance data within cecal content group are less different than that of feces samples (Figure 3) [19]. This suggests that future metabolite profiling studies would require fewer samples of colonic-cecal contents than that of feces within a treatment group.

**Figure 4:**
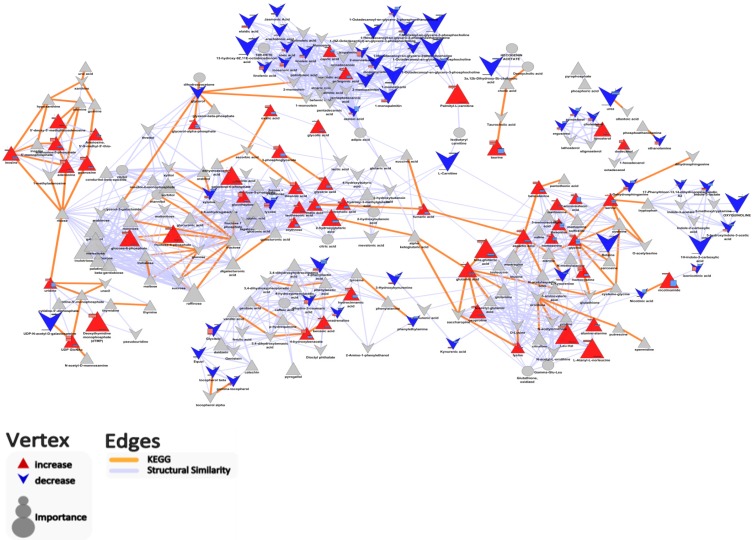
Biochemical and chemical similarity network highlighting important differences in metabolites abundance between colonic-cecal content and feces. Vertex size encodes the fold change in metabolite abundance between groups (when the metabolite abundance in colonic-cecal content was higher than that in feces, we defined it as “increase”; in contrast, if the metabolite abundance in colonic-cecal content was lower than that in feces, we defined it as “decrease”). Vertex shape (triangle, increase; “V”, decrease; ellipse, no change) and color (blue, decrease; red, increase; gray, insignificant change or p > 0.05 after-adjustment for FDR) are used to encode the significance and relative direction of changes in metabolites between colonic-cecal content and feces comparison.

Biochemical network analysis (defined by KEGG) revealed that the abundance of most carbohydrate metabolites (e.g., galactose-6-phosphase, erythrose), and amino acid metabolites (e.g., glutamic acid, aspartic acid, leucine, valine) were greater in colonic cecal contents than that in feces (Figure 4, Table S1). This is in agreement with the fact that complex carbohydrates and poorly digested proteins may reach the hind gut to be available for colonic bacterial fermentation [11,12,13,14,20]. In contrast, the contents of most fatty acid-related metabolites (e.g., linoleic acid, jasmonic acid, and phospholipid) were greater in feces than in colonic-cecal contents (Figure 4, Table S1). The higher content of phospholipid in feces may reflect bacterial breakdown [12,21]. 

Collectively, the metabolite detection, unique metabolites, relative abundance comparison and network analyses are the key methods to study the gut metabolome in our future nutritional intervention experiments.

## 3. Experimental Section

### 3.1. Animals, Diets, and Treatment

This study was approved by the Animal Care and Use Committee of the Grand Forks Human Nutrition Research Center, and animals were maintained in accordance with NIH guidelines for the care and use of laboratory animals. Male C57BL/6 mice, 5 months old and body weight (30 g), were individually housed in Plexiglas™ ventilated cages within a pathogen-free facility that maintained a 12-h light/dark cycle. Mice (n = 5) were given free access to deionized water, and a common practical-type, chow diet (LabDiet cat#5015, St. Louis, MO). This chow diet is a complete life cycle diet, in which 19.8% calories from protein, 26.1% calories from fat (ether extract) and 54.1% calories from carbohydrates. These mice had been in this diet for 5 months (their entire life time). At the termination of the experiment, mice were euthanized with a mixture of ketamine and xylazine by intraperitoneal injection. Feces were collected over a 24-h period prior to euthanasia. After euthanasia, the gut was prepared by dissection, the colonic and cecal contents were combined, and all samples were placed in sealed containers and held at −80 °C prior to analysis.

### 3.2. Sample Extraction

Aliquots (10 mg) of colonic-cecal contents and feces were each thawed and extracted by agitation with 1mL of degassed acetonitrile: isopropanol: water (3:3:2) at –20 °C after which the soluble portion was recovered by centrifugation. Aliquots (450 µL) of that supernate were used for each GC-TOF and LC-Q-TOF analyses.

### 3.3. GC-TOF Analysis

Extracts were derivatized as previously published [22], and then analyzed using an Agilent 7890A gas chromatograph (Santa Clara, CA) coupled to a Leco Pegasus IV time-of-flight mass spectrometer. A Gerstel MPS2 automatic liner exchange system (ALEX) was used to eliminate sample cross-contamination during the GC-TOF analysis. The column was 30 m, 0.25 mm i.d. Rtx5Sil-MS with 0.25 µm 5% diphenyl film with a 10 m integrated guard column (Restek, Bellefonte PA) [23,24,25]. The sample (0.5 µL) was injected at 50 °C ramped to 250 °C in splitless mode with a 25 s splitless time. The chromatographic gradient consisted of a constant flow of 1 ml/min, ramping the oven temperature from 50 °C for to 330 °C over 22 min. Mass spectrometry parameters were: 280 °C transfer line temperature, electron ionization at −70 V, and a 250 °C ion source temperature. Mass spectra were acquired at 1525 V detector voltage at *m/z* 85–500 with 17 spectra/sec. Acquired spectra were further processed using the BinBase database [25,26,27]. Data quality and instrument performance were monitored throughout the data acquisition using quality control and reference plasma samples (NIST), as previously described [22].

### 3.4. LC-Q-TOF Data Collection and Analysis

Extracts were evaporated to dryness under reduced pressure, and reconstituted in 50 µL of water:acetonitrile (98:2 v:v), sonicated (5 min) and, then, centrifuged. Supernates (40 µL) were transferred to amber glass vials (National Scientific-C4000-2W) fitted with micro-inserts (Supelco 27400-U).

Polar extracts were analyzed using an Agilent 1290 A Infinity Ultra High Performance Liquid Chromatography system coupled to an Agilent Accurate Mass-6530-QTOF in both positive and negative modes. The column (40 °C) was a Waters Acquity BEH Shield RP18 Column (150 mm length x 2.1 mm internal diameter; 1.7 µM particles). Mobile Phase A consisted of 100% LCMS grade water containing 0.1% formic acid; mobile phase B consisted of 100% acetonitrile containing 0.1% formic acid. The gradient started from 0 min 2% (B), 0–0.5 min 2% (B), 0.5–12 min 95% (B), 12–12.5 min 95% (B), 12.5–12.6 min 2% (B), and 12.6 min–17 min 2% (B). The flow rate was 0.6 mL/min; an injection volume was 5 µL for both ESI (+/−) mode acquisitions with an ESI capillary voltage was + 3.5 kV and −3.5 kV for positive and negative mode, respectively. For MSMS, collision energies were 10, 20 and 40 eV for both positive and negative acquisition modes. Data were collected over a mass range of m/z 60–1200 Da with a spectral acquisition speed of 2 spectra per sec.

Data were processed using MZmine 2.10. All peak intensities are representative of peak heights. Annotations were completed by matching experimental accurate mass MS/MS spectra to MS/MS libraries, including Metlin-MSMS, NIST12, and LipidBlast. Spectral matching was accomplished using MSPepSearch manually curated using The NIST Mass Spectral Search Program Version 2.0g. Metabolite libraries were created, in positive and negative ionization modes, containing all confirmed identified compounds. MZmine’s Custom Database Search tool was used to assign annotations based on accurate mass and retention time matching.

### 3.5. Data Analysis

Data collected by GC-TOF and LC-Q-TOF were merged for both types of sample. GC-TOF values are reported for metabolic features measured on both platforms with analysis limited to annotated features. Missing values for the LC-Q-TOF were imputed as zeros. Statistical analyses were conducted on natural logarithm-transformed metabolic parameters, and data summaries are presented for raw values. Multivariate analyses (clustering, PCA, O-PLS-DA) were implemented on natural logarithm-transformed and auto-scaled values (mean centered and scaled by the standard deviation, z-scaled).

Statistical analyses were implemented in R v3.01. Independent sample t-Tests were used to test for differences in metabolite profiles between colonic-cecal contents and feces. The probability or significance level for the test statistics (*i.e.*, p-values) were adjusted for the multiple hypotheses tested [28], and the false discovery rate (FDR)-adjusted p-values are reported as “p_adj_”. The FDR was also directly estimated as the q-value, for all comparisons [29]. Hierarchical cluster analysis (HCA) was used to group samples and metabolites based on similarities in auto-scaled values and correlations, respectively [6]. Principal components analysis [6] was used to evaluate the sample class structure including similarities and differences. Orthogonal signal correction partial least squares discriminant analysis (O-PLS-DA) [18] was used to develop a multivariate classification model for the two types of samples. In order to visualize the biological interrelationship of the 270 known metabolites in colonic cecal contents and feces, biochemical and chemical similarity network analysis was used to generate networks vertices based on biochemical relationships (KEGG RPAIR Database).

## 4. Conclusions

In summary, we have conducted the first comprehensive comparison of the metabolite profiles of colonic-cecal contents and feces in the mouse. Our results show that these specimens have very similar metabolite profiles, indicating the suitability of feces, which can be sampled non-invasively, as proxies for estimating the metabolite patterns of hindgut microbiome. These findings should inform future research on the role of diet and the gut microbiome in supporting health.

## Supplementary Files

Supplementary File 1

## References

[1] Cox A.J., West N.P., Cripps A.W. (2015). Obesity, inflammation, and the gut microbiota. Lancet Diabetes Endocrinol..

[2] Akin H., Tözün N. (2014). Diet, microbiota, and colorectal cancer. J. Clin. Gastroenterol..

[3] Moreno-Indias I., Cardona F., Tinahones F.J., Queipo-Ortuño M.I. (2014). Impact of the gut microbiota on the development of obesity and type 2 diabetes mellitus. Front Microbiol..

[4] Dettmer K., Aronov P.A., Hammock B.D. (2007). Mass spectrometry-based metabolomics. Mass Spectrom. Rev..

[5] Scalbert A., Brennan L., Fiehn O., Hankemeier T., Kristal B.S., van Ommen B., Pujos-Guillot E., Verheij E., Wishart D., Wopereis S. (2009). Mass-spectrometry-based metabolomics: limitations and recommendations for future progress with particular focus on nutrition research. Metabolomics.

[6] Sugimoto M., Kawakami M., Robert M., Soga T., Tomita M. (2012). Bioinformatics Tools for Mass Spectroscopy-Based Metabolomic Data Processing and Analysis. Curr. Bioinform..

[7] Creek D.J., Jankevics A., Burgess K.E., Breitling R., Barrett M.P. (2012). IDEOM: an Excel interface for analysis of LC-MS-based metabolomics data. Bioinformatics.

[8] Kanehisa M. (2002). The KEGG database. Novartis Found Symp..

[9] Nyangale E.P., Mottram D.S., Gibson G.R. (2012). Gut microbial activity, implications for health and disease: the potential role of metabolite analysis. J. Proteome Res..

[10] Zeng H., Lazarova D.L., Bordonaro M. (2014). Mechanisms linking dietary fiber, gut microbiota and colon cancer prevention. World J. Gastrointest. Oncol..

[11] Evenepoel P., Geypens B., Luypaerts A., Hiele M., Ghoos Y., Rutgeerts P. (1998). Digestibility of cooked and raw egg protein in humans as assessed by stable isotope techniques. J. Nutr..

[12] Macfarlane G.T., Macfarlane S. (2012). Bacteria, colonic fermentation, and gastrointestinal health. J. AOAC. Int..

[13] Nordgaard I., Mortensen P.B. (1995). Digestive processes in the human colon. Nutrition.

[14] Russell W.R., Hoyles L., Flint H.J., Dumas M.E. (2013). Colonic bacterial metabolites and human health. Curr. Opin. Microbiol..

[15] Yahiro K., Setoguchi T., Katsuki T. (1980). Effect of cecum and appendix on 7 alpha-dehydroxylation and 7 beta-epimerization of chenodeoxycholic acid in the rabbit. J. Lipid Res..

[16] Clarke G., Stilling R.M., Kennedy P.J., Stanton C., Cryan J.F., Dinan T.G. (2014). Minireview: Gut microbiota: the neglected endocrine organ. Mol. Endocrinol..

[17] Bergkvist A., Rusnakova V., Sindelka R., Garda J.M., Sjögreen B., Lindh D., Forootan A., Kubista M. (2010). Gene expression profiling—Clusters of possibilities. Methods.

[18] Wehrens R. (2011). Chemometrics with R: Multivariate Data Analysis in the Natural Sciences and Life Sciences.

[19] Okada T., Afendi F.M., Altaf-Ul-Amin M., Takahashi H., Nakamura K., Kanaya S. (2010). Metabolomics of medicinal plants: the importance of multivariate analysis of analytical chemistry data. Curr. Comput. Aided Drug Des..

[20] Barcenilla A., Pryde S.E., Martin J.C., Duncan S.H., Stewart C.S., Henderson C., Flint H.J. (2000). Phylogenetic relationships of butyrate-producing bacteria from the human gut. Appl. Environ. Microbiol..

[21] Ramakrishna B.S. (2013). Role of the gut microbiota in human nutrition and metabolism. J. Gastroenterol. Hepatol..

[22] Fiehn O., Wohlgemuth G., Scholz M., Kind T., Lee do Y., Lu Y., Moon S., Nikolau B. (2008). Quality control for plant metabolomics: Reporting MSI-compliant studies. Plant J..

[23] Weckwerth W., Wenzel K., Fiehn O. (2004). Process for the integrated extraction, identification and quantification of metabolites, proteins and RNA to reveal their co-regulation in biochemical networks. Proteomics.

[24] Fiehn O. (2008). Extending the breadth of metabolite profiling by gas chromatography coupled to mass spectrometry. Trends Analyt. Chem..

[25] Kind T., Tolstikov V., Fiehn O., Weiss R.H. (2007). A comprehensive urinary metabolomic approach for identifying kidney cancerr. Anal. Biochem..

[26] Fiehn O., Wohlgemuth G., Scholz M. (2005). Setup and annotation of metabolomic experiments by integrating biological and mass spectrometric metadata. Data intergration in the life Sciences, Proceedings of Second International Workshop, DILS 2005.

[27] Scholz M., Fiehn O. (2007). SetupX—a public study design database for metabolomic projects. Pac. Symp. Biocomput..

[28] Benjamini Y., Hochberg Y. (1995). Controlling the false discovery rate—a practical and powerful approach to multiple testing. J. Roy. Stat. Soc. B Met..

[29] Klaus B., Strimmer K. (2012). fdrtool: Estimation of (local) false discovery rates and higher criticism.

